# Exploring the mechanism of action of aspirin in improving endometrial receptivity in PCOS rats based on uterine lavage fluid metabolomics

**DOI:** 10.1371/journal.pone.0324432

**Published:** 2025-05-22

**Authors:** Ying Zhang, Xi Wang, Yiqing He, Quan Liu, Shuo Yang

**Affiliations:** Hunan University of Chinese Medicine, Changsha, Hunan, China; Shiraz University of Medical Sciences, IRAN, ISLAMIC REPUBLIC OF

## Abstract

**Backgrounds:**

Aspirin has been shown to enhance endometrial receptivity (ER) during the window of implantation in patients with polycystic ovary syndrome (PCOS). However, the underlying mechanisms remain unclear. This study aimed to elucidate the mechanisms by which aspirin improves ER through metabolic analysis of uterine lavage fluid.

**Methods:**

A PCOS rat model was established using letrozole. Body weight and estrous cycles were monitored, and the number of implanted embryos was assessed across groups. We evaluated endometrial ultrastructure, ovarian and endometrial histomorphometry. Serum levels of estradiol(E_2_) and progesterone(P)were measured. Moreover, through ultra-performance liquid chromatography-mass spectrometry, the study of uterine lavage fluid metabolites revealed the potential mechanism of action of aspirin.

**Results:**

Compared with the model group, aspirin treatment significantly increased embryo implantation rates, improved endometrial morphology and hormone levels. Metabolomic analysis identified 48 differential metabolites, among which five—2, 6-dihydroxypurine, gluconolactone, Oxaceprol, PC (18:1/18:1), and PC (20:3e/17:1)—were identified as potential biomarkers for aspirin-mediated improvement of ER in PCOS rats. Pathway analysis revealed that aspirin primarily modulates the pentose phosphate pathway, arginine and proline metabolism, and glycerophospholipid metabolism.

**Conclusions:**

Aspirin may enhance glucose metabolism, alleviate insulin resistance, promote angiogenesis, and improve vascular permeability and endometrial receptivity. These effects are likely mediated through the regulation of biomarkers involved in the pentose phosphate pathway, arginine and proline metabolism, and glycerophospholipid pathways in uterine lavage fluid.

## Introduction

Polycystic ovarian syndrome (PCOS), a prevalent reproductive endocrine disorder affecting 10–13% of women in their reproductive age, is characterized by hyperandrogenemia, ovulatory dysfunction, polycystic ovarian morphology, and often by associated metabolic disorders [1]. As is known to all, endometrial receptivity of PCOS was regarded as the one of the causes of low pregnancy rate and high abortion rate [2]. Despite the high prevalence of PCOS, pharmacologic interventions for such a complicated syndrome encounter substantial challenges. There are limited options available to improve endometrial receptivity in patients with PCOS. Therefore, proven therapeutic strategies are urgently needed.

Aspirin, a widely utilized nonsteroidal anti-inflammatory drug (NSAID) [3]. Low-dose aspirin has been employed for its anti-inflammatory, vasodilatory, and platelet aggregation-inhibitory effects to enhance blood flow, support fertility, and improve the success rates of in vitro fertilization (IVF) therapy. These characteristics, combined with its low cost, widespread availability, and favorable safety profile at low doses, render aspirin a promising adjunct for women undergoing ovulation induction therapy [4]. Numerous studies have demonstrated that low-dose aspirin significantly enhances uterine perfusion and improves pregnancy outcomes in IVF patients by promoting ER [5,6]. Therefore, we regard that aspirin may be a potential therapeutic agent for improving ER. However, the specific mechanisms through which aspirin modulates ER, especially in PCOS, remain poorly understood.

Recent studies suggest that uterine fluid collection may be proposed to become a less-invasive routine practice compared to endometrial biopsy, avoiding the risk of endometrial damage [7]. Moreover, the relationship between uterine lavage fluid metabolites and ER in PCOS models remains under-explored. Therefore, in this study, we performed an un-targeted metabolomic analysis using ultra-high-performance liquid chromatography-tandem mass spectrometry (UHPLC-MS/MS) to investigate the effects of aspirin on uterine lavage fluid metabolites in PCOS rats during the window of implantation. Our primary objective was to identify potential biomarkers associated with aspirin-induced improvement in endometrial receptivity (ER) and to elucidate the underlying metabolic pathways involved. Based on our findings, we hypothesized that aspirin improves ER in PCOS rats by modulating key metabolic pathways, such as the pentose phosphate pathway, arginine and proline metabolism, and glycerophospholipid metabolism, thereby enhancing endometrial morphology, angiogenesis, and pinopodes development, and consequently, promotes embryo implantation. Thereby providing a foundational understanding of aspirin's role and underlying mechanisms in improving ER.

## Materials and methods

### Chemicals and reagents

Letrozole (211026KL) was provided by Jiangsu Hengrui Medical Co., Ltd. (Jiangsu, China). Aspirin enteric-coated tablets (210503) from Hunan Xinhui Pharmaceutical Co. Wright staining (G1040) was purchased from Beijing Solarbio Science and Technology Co., Ltd. (Beijing, China). Hematoxylin and eosin (HE) staining solution (ZLI-1040、ZLI-9610) was bought from Beijing Zhongshan Jinqiao Biotechnology Co. (Beijing, China). The standards of HPLC grade ultra-pure water, methanol, formic acid, and ammonium acetate (7732-18-5, 67–561, 64-18-6, 631-61-8) were bought from Thermo Fisher (USA). Estradiol (E_2_) (ML-E202302731) and progesterone (P) (ML-E202302225) of the enzyme-linked immunosorbent assay (ELISA) kits were all acquired from Shanghai Fusheng Industrial Co. Ltd. (Shanghai, China). The electron microscope fixative and PBS were purchased from Wuhan Xavier Biotechnology Co.

### Animals and induction of PCOS

Eighteen female Sprague Dawley (SD) rats (weight 190-210g, 6–8 weeks old) and nine sexually mature male SD rats (weight 340-360g, 13–15 weeks old) were brought from Hunan SJA Laboratory Animal Co, Ltd. (Animal certificate number: SCXK (Xiang) 2019–0004). All rats were maintained in a light (06:00–18:00)-dark (18:00–6:00) cycle with free food. The control temperature was 22 °C ± 2 °C, and humidity was 50 ± 10% in the Laboratory Animal Center of Hunan University of Chinese Medicine. All animal experiments were approved by the Ethics Committee of Hunan University of Chinese Medicine (ethics approval number: LL2022031603). The disposal of animals complied with The Guiding Opinions on Treating Laboratory Animals Kindly published by the Ministry of Science and Technology of China.

The rats were acclimatized and fed for 7 days. Vaginal smears of the rats were taken to determine the estrous cycle at 7:00–8:00 a.m. daily [8]. Eighteen rats were divided into a control group (n = 6) and a model group (n = 12). The control group received a normal diet, whereas the model group received letrozole (1 mg kg^−1^ day^−1^, dissolved in 0.5% carboxymethyl cellulose (CMC-Na) for 21 consecutive days to establish the PCOS model [9]. The rats showed disturbed, prolonged or even stagnant estrous cycle, and the histopathological section of the ovary showed polycystic changes, suggesting that the modeling was successful. The PCOS rats were divided into the model group (n = 6) and the Aspirin group (n = 6). The rats in the aspirin group were given intragastric administration of aspirin (8 mg kg^−1^ day^−1^, dissolved in 0.5% CMC-Na). Meanwhile, the rats of the control and model groups were given the same volume of saline as the Aspirin group rats based on their individual body weight.

### Experimental design

Pharmacological intervention in rats in the aspirin group lasted for 12 days. On the day of the last drug administration, the cages were combined according to the ratio of male to female of 2:1. Every morning, female rats were observed for vaginal plugs and vaginal smears for spermatozoa, with the observation of vaginal plugs or vaginal smears for spermatozoa recorded as day 1 of gestation, and the samples were taken on day 5 of gestation. Deep anesthesia was induced by intraperitoneal injection of 2% sodium pentobarbital to ensure that the rats remained pain-free throughout the procedure. The depth of anesthesia was assessed by the absence of corneal reflexes and pain stimulus responses. After confirming complete anesthesia, euthanasia was performed by exsanguination via abdominal aorta puncture. Blood was collected until confirmed cardiac arrest occurred, which was verified by cessation of heartbeat and respiration for at least 5 minutes.Blood samples were centrifuged at 3000 r/min for 15 minutes, and the supernatants were obtained for sex hormone level tests [10]. Histopathology was performed on one side of each rat's ovaries and treated with a 4% paraformaldehyde buffer [11]. The uterus was separated bilaterally and the bicornuate uterus was removed. Repeatedly rinse the uterine cavity with 2 ml of sterilized ultra-pure water and the fluid was collected and placed in a lyophilization tube. The samples were stored in liquid nitrogen and finally in the –80°C refrigerator. The uterus was dissected, and the number of follicle implantation was recorded. One fourth of the uterine tissue was fixed in 4% paraformaldehyde, embedded in paraffin, and sliced for HE detection [11]. Another 5 mm^3^ of uterine tissue was fixed in the electron microscope fixative for scanning electron microscopy [12].

### Detection of uterine lavage fluid metabolites by mass spectrometry

#### Sample pretreatment.

Remove the sample of uterine lavage fluid from the refrigerator at -80°C, thaw at 4°C. Uterine lavage fluid (100 μL) was placed in the Eppendorf (EP) tubes and re-suspended with pre-chilled 80% methanol (400 μL) by the well vortex. After 5 min on ice, the samples were centrifuged at 15,000 × g, 4 °C for 20 mins. The supernatant (400 μL) was diluted to a final concentration containing 53% methanol by LC-MS grade water. The samples were transferred to a fresh EP tube and centrifuged at 15,000 × g, 4°C for 20 mins. Finally, the supernatant was injected into the LC-MS/MS system. The quality control (QC) sample was made by combining equal quantities of each sample and 30 μL of supernatant as part of the quality control and system conditioning process [13].

#### UHPLC-MS/MS conditions.

The UHPLC separation was performed using a Vanquish UHPLC system (Thermo Fisher, Germany) equipped with a Hypersil GOLD column (100 × 2.1 mm, 1.9 μm). The positive polarity mode eluents consisted of 0.1% formic acid in water (A) and methanol (B). The eluents of the negative polarity mode were eluent A (5 mM ammonium acetate, pH 9.0) and eluent B (methanol). Gradient elution of liquid chromatography at the flow rate of 0.2 mL/min was set as follows: 2% B, 1.5 min; 2–85% B, 3 min; 85–100% B, 10 min; 100–2% B, 10.1 min; and 2% B, 12 min. AQ Exactive TM HF-X mass spectrometer (Thermo Fisher, Germany) provided with an electrospray ionization source was used to obtain the mass spectrometric data. Ion source parameters were as follows: spray voltage of 3.5 kV, capillary temperature of 320 °C, sheath gas flow rate of 35 psi, aux gas flow rate of 10 L/min, S-lens RF level of 60, and aux gas heater temperature of 350°C. Polarity: positive, negative. negative; MS/MS secondary scans were data-dependent scans [14].

#### Multivariate data processing and analysis.

Compound Discoverer 3.1 (CD3.1, Thermo Fisher) was used to align, identify, and quantify metabolites from UHPLCMS/MS data. These metabolites were annotated using the Kyoto Encyclopedia of Genes and Genomes (KEGG) database (https://www.genome.jp/kegg/pathway.html) and the Human Metabolome Database (HMDB) (https://hmdb.ca/metabolites). For the multivariate statistical analysis part, the data were transformed using metabolomics data processing software metaX and then subjected to principal component analysis (PCA) and partial least squares discriminant analysis (PLS-DA) to obtain the VIP value for each metabolite [15]. We applied univariate analysis (t-test) to calculate the statistical significance (P-value). A threshold value of VIP > 1.0 and P < 0.05 was set for screening the metabolite, in which VIP values stand for variable influence on projection (VIP) and P-values represent statistical significance [16]. Statistical analyses were performed using the statistical software R (R version R-3.4.3), Python (Python 2.7.6 version), and CentOS (CentOS release 6.6). When data were not normally distributed, normal transformations were attempted using the area normalization method.

### Statistical analysis

All data were expressed as mean ± Standard Deviation (SD) and analyzed by SPSS 27.0 statistical analysis software. The one-way ANOVA was used for multiple group comparisons, and the least significant difference method LSD test was used for two-by-two comparisons; for non-normally distributed data, Kruskal-Wallis test was used A value of *P* < 0.05 is considered statistically significant. Graphs were generated using GraphPad Prism 9.5.0 software.

## Results

### Determination of PCOS rat model

**Fig 1:**
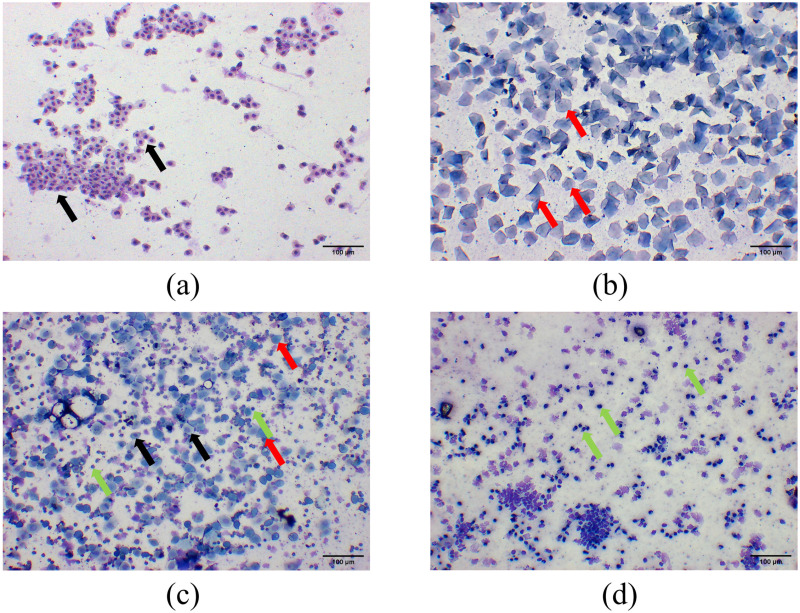
Changes in the estrous cycle of PCOS rats (Wright’s staining, scare bar = 100 μm). **(a–d)** Vaginal smears corresponding to different phases of the estrous cycle: **(a)** Proestrus, (P) was dominated by expanded oval-shaped nucleated epithelial cells (black arrow); **(b)** Estrus **(E)**, estrus was dominated by irregularly shaped anucleate keratinized cells(red arrow); **(c)** Metestrus **(M)**, metestrus was characterized by leukocytes(green arrow), nucleated epithelial cells (black arrow) and keratinized cells(red arrow) in comparable quantities; **(d)** Diestrus **(D)**, diestrus was mainly dominated by leukocytes(green arrow).

**Fig 2:**
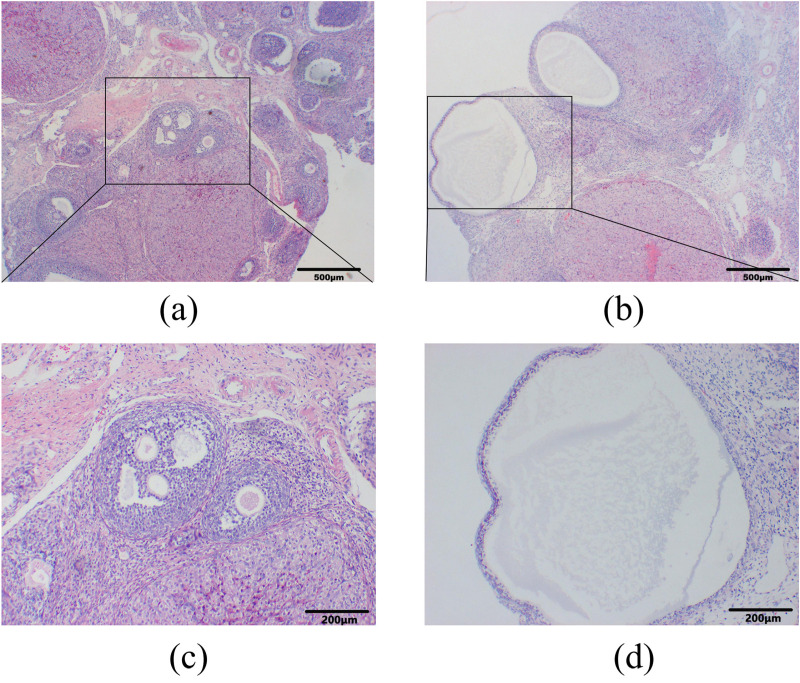
Morphological changes in ovarian tissue of PCOS rats. **(a, c)** Control group, the oocyte structure was intact and clear, the granulosa cells were multilayered, tightly and neatly arranged, and multiple corpus luteum and follicles at all levels were visible; **(b, d)** Model group, a large number of cystic follicles were visible in the ovary, the number of oocytes and corpus luteum was significantly reduced, and the number of granulosa cell layers was reduced and loosely arranged. The lower panels (HE staining, scare bar = 200μm) show magnified views of the boxed regions in the upper panels (HE staining, scare bar = 500μm).

The pre-motility period (proestrus, P) was dominated by expanded oval-shaped nucleated epithelial cells; the estrus period (estrus, E) was dominated by irregularly shaped anucleate keratinized cells; the metestrus period (metestrus, M) was characterized by leukocytes, nucleated epithelial cells and keratinized cells in comparable quantities; and the inter-estrus period (diestrus, D) was mainly dominated by leukocytes. The results of estrous cycle monitoring showed that the rats in the control group had a regular estrous cycle, while the rats in the model group had a disorganized estrous cycle with a prolonged cycle, or even stagnation persisting in the inter-estrous phase, and the estrous cycle lost its regularity (Fig 1). Sections of ovarian tissue showed that in the control group, the oocyte structure was intact and clear, the granulosa cells were multilayered, tightly and neatly arranged, and multiple corpus luteum and follicles at all levels were visible; in the model group, a large number of cystic follicles were visible in the ovary, the number of oocytes and corpus luteum was significantly reduced, and the number of granulosa cell layers was reduced and loosely arranged (Fig 2). The rats showed disturbed, prolonged or even stagnant estrous cycle, and the histopathological section of the ovary showed polycystic changes, suggesting that the modeling was successful.

### Effect of aspirin on blastocyst implantation in PCOS rats during the window of implantation

**Fig 3:**
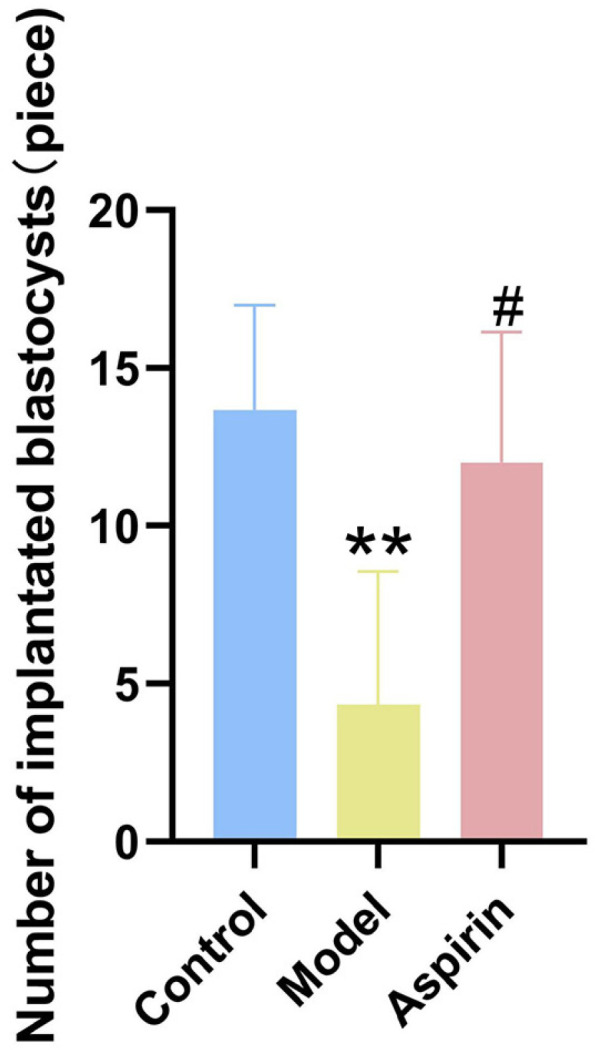
Effect of aspirin on the number of blastocysts implanted in PCOS rats during the window of implantation. (n = 6). **P ＜ 0.01 compared with the control group, and ^#^*P* ＜ 0.05compared with the model group.

The number of implanted blastocysts was significantly reduced in the model group compared to the control group (*P* < 0.01). In contrast, aspirin treatment led to a significant increase in the number of implanted blastocysts compared to the model group (*P* < 0.05). These findings suggest that aspirin effectively enhances blastocyst implantation in PCOS rats during the window of implantation (Fig 3).

### Effect of aspirin on serum E_2_ and P levels in PCOS rats during the window of implantation

**Fig 4:**
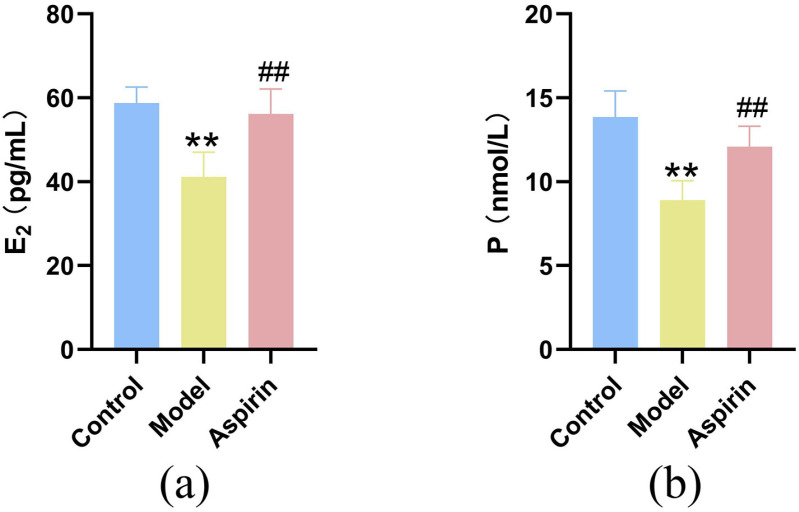
Effect of aspirin on serum E_2_ and P levels in PCOS rats during the window of implantation. (n = 4). **(a)** Serum E_2_ level; **(b)** Serum P level. **P ＜ 0.01 compared with the control group, and ^##^*P* ＜ 0.01 compared with the model group.

Serum levels of estradiol (E_2_) and progesterone (P) were significantly lower in the model group compared to the control group (*P* < 0.01). In contrast, aspirin treatment significantly increased the levels of E_2_ and P compared to the model group (*P *< 0.01), suggesting that aspirin effectively promotes the elevation of E_2_ and P levels in the serum of PCOS rats (Fig 4).

### Effects of aspirin on endometrial histomorphology in PCOS rats during the window of implantation

**Fig 5:**
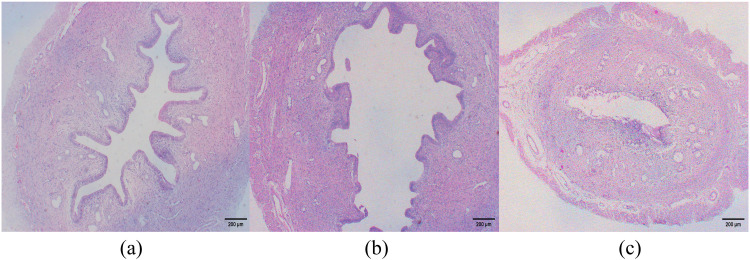
Effect of aspirin on endometrial histomorphometry in PCOS rats during the window of implantation. (n = 5). (HE staining, scare bar = 200μm). **(a)** Control group; **(b)** Model group; **(c)** Aspirin group.

**Fig 6:**
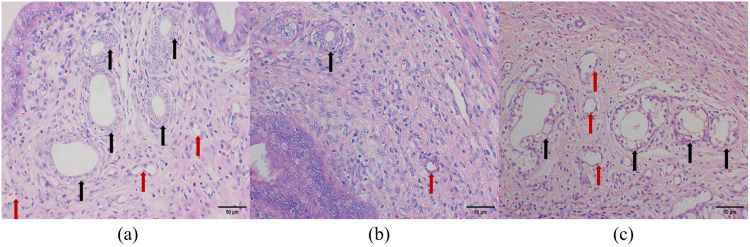
Effect of aspirin on endometrial histomorphometry in PCOS rats during the window of implantation. (n = 5). (HE staining, scare bar = 50μm). **(a)** Control group, the endometrial stroma is loose, with synchronous development of glands and stroma, abundant glands, and a rich vascular supply; **(b)** Model group, endometrial glandular hypoplasia is observed, characterized by small glandular lumina and a reduced number of glands, along with a decrease in vascularity; **(c)** Aspirin group, endometrial development shows improvement, manifested by larger glandular lumina, loose stroma, and an increased number of glands and vessels within the stroma. Black arrows indicate glands, and red arrows indicate blood vessels.

**Fig 7:**
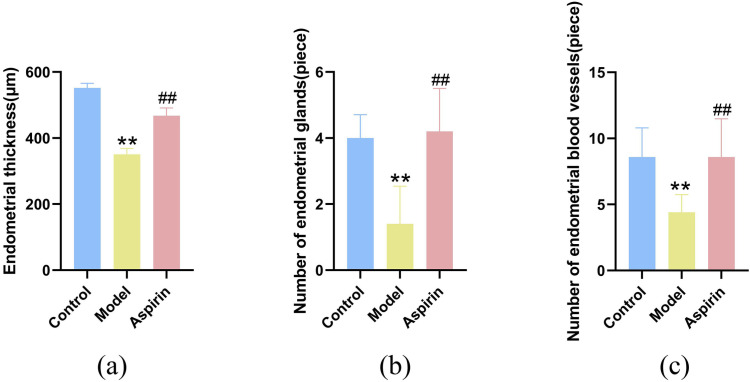
Effect of aspirin on endometrial histomorphology in PCOS rats during the window of implantation. (n = 5). **(a)** Endometrial thickness; **(b)** Number of endometrial glands; **(c)** Number of endometrial blood vessels. **P ＜ 0.01 compared with the control group, and ^##^*P* ＜ 0.01 compared with the model group.

Compared to the control group, the model group exhibited significantly reduced endometrial thickness, along with a marked decrease in the number of endometrial glands and blood vessels (*P* < 0.01). In contrast, aspirin treatment significantly increased endometrial thickness and the number of endometrial glands and blood vessels compared to the model group (*P* < 0.01). These findings suggest that aspirin effectively enhances endometrial thickness, promotes glandular development, and stimulates angiogenesis (Figs 5–7).

### Effect of aspirin on the expression of pinopodes in PCOS rats during the window of implantation

**Fig 8:**
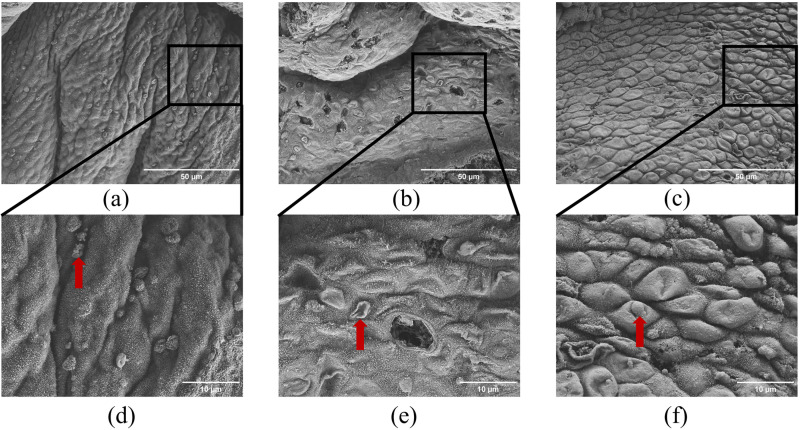
Effect of aspirin on the expression of pinopodes in PCOS rats during the window of implantation. (n = 3). **(a, d)** Control group, the endometrial architecture appeared normal and intact, exhibiting a dense and uniform distribution of microvilli and numerous pinopodes were observed, evenly distributed across the surface; **(b, e)** Model group, the endometrial structure showed significant disruption, characterized by sparse and irregularly distributed microvilli and the number of pinopodes was markedly reduced, with the majority exhibiting pronounced wrinkling and collapse; **(c, f)** Aspirin group, the endometrial structure was slightly damaged, with abundant and evenly distributed microvilli and a large number of pinopodes, but some of them were slightly wrinkled. The lower panels (scare bar = 10μm) magnify the boxed regions in the upper panels (scare bar = 50μm). Red arrows indicate pinopodes.

Scanning electron microscopy was used to observe changes in the morphology and number of pinopodes in the endometrium of PCOS rats. In the control group, the endometrial structure appeared normal and intact, with numerous pinopodes and abundant, uniformly distributed microvilli. In contrast, the model group exhibited severe endometrial damage, with a significant reduction in the expression of pinopodes. Most of these synapses were severely crumpled and collapsed, and microvilli were sparse and unevenly distributed. Compared to the model group, the aspirin group demonstrated partial repair of endometrial damage, with an increased expression of pinopodes and more abundant, evenly distributed microvilli. These findings suggest that aspirin can improve the expression of pinopodes in the endometrium of PCOS rats during the window of implantation, and thus promote embryo adhesion (Fig 8).

### Effect of aspirin on the metabolic profile of uterine lavage fluid in PCOS rats during the window of implantation

#### UHPLC-MS/MS stability analysis.

Metabolic profiling of uterine lavage fluid from PCOS rats revealed comparable total ion chromatograms (TICs) for the control, model, and aspirin groups, although differences in the response values of the peaks were observed. TICs, including retention time (RT), peak intensity, and resolution, were recorded for five quality control (QC) samples in both positive and negative ion modes using UHPLC-MS/MS. Pearson correlation coefficients between the QC samples were calculated based on the relative quantitative values of the metabolites. A higher correlation between the QC samples (with R² closer to 1) indicates better assay stability and higher data quality.

#### Multivariate statistical analysis.

**Fig 9:**
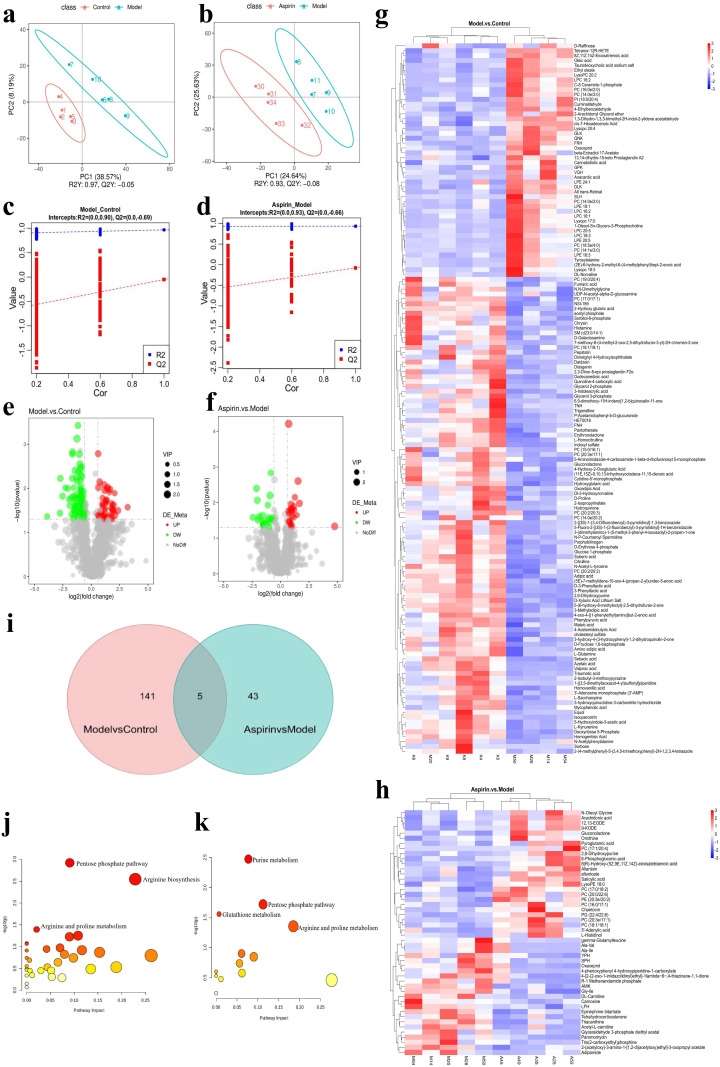
Multivariate statistical analysis diagram. **(a, b)** PLS-DA diagrams. The separation pattern was satisfactory, with significant differences in principal components among the groups, indicating that the metabolic profiles underwent significant alterations between different groups. The ellipses represent the 95% confidence intervals. **(c, d)** 200 × permutation test of 7-fold cross-validation. The PLS-DA model was validated by a 200 × permutation test, and the results were R^2^Y = 0.90, Q^2^ = -0.69 < 0 and R^2^Y = 0.93, Q^2^ = -0.66 < 0. The model is well established, there is no over-fitting. **(e, f)** Volcano plots. The volcano plots showed the changing trend of the differential metabolites expression in the Model group vs. Control group and Aspirin group vs. Model group. Each dot represents a specific metabolite, and the size of the dot indicates the VIP value. Red dots represent upregulated metabolites; green dots represent down-regulated metabolites. **(g, h)** Heat maps of metabolites. In the form of a heat map, shows a full range of metabolites in uterine lavage fluid samples from the Model group vs. Control group and Aspirin group vs. Model group and the relative expression magnitude of metabolites in each sample group. Red indicates increased biomarker expression; blue indicates decreased biomarker expression. **(i)** Venn diagram of metabolites. Five common cross metabolites were screened by intersecting the metabolites in the Model group vs. Control group and Aspirin group vs. Model group. **(j, k)** Metabolite pathways. Large size and red color represent major pathway enrichment and high pathway effect values, respectively. Statistically significant pathways (*P* < 0.05) have been labelled. **(j)** Model group vs. Control group; **(k)** Aspirin group vs. Model group.

Partial Least Squares Discrimination Analysis (PLS-DA) was employed to model the relationship between metabolite expression and sample categories, revealing significant metabolic differences between the groups. The analysis demonstrated clear distinctions in metabolites between the control and model groups, as well as between the model and aspirin groups of uterine lavage fluid (Fig 9.a–b). To assess whether over-fitting occurred in the PLS-DA model, a 200 × permutation test was performed. The results indicated that for the model group vs. the control group, the R²Y was 0.90, and Q² was -0.69; for the aspirin group vs. the model group, the R² Y was 0.93, and Q² was -0.66. The Q² regression lines intercept on the model's longitudinal axis was below zero, suggesting that the model was well-constructed without over-fitting and is reliable for further analysis (Fig 9.c–d). These results confirm the validity of the model and its suitability for differential metabolite screening. Mars plots displayed trends of differential metabolite expression between the model and control groups, and between the aspirin and model groups (Fig 9.e–f). In these plots, each dot represents a specific metabolite, with dot size reflecting the Variable Importance in Projection (VIP) value. Larger dots indicate higher VIP values, while green dots represent down-regulated metabolites, and red dots represent up-regulated metabolites.

#### Identification of potential biomarkers.

Based on the PLS-DA results, *P* < 0.05 and VIP > 1.0 were considered as significance thresholds, which were used as criteria to screen for differential metabolites. A total of 146 significantly different metabolites were identified in the comparison between the model group and the control group, with notable changes following aspirin intervention. In the comparison between the aspirin and model groups, 48 significantly different metabolites were found. Compared with the control group, 48 metabolites were up-regulated and 98 were down-regulated in the model group (*P* < 0.05); in the aspirin group, 25 metabolites were up-regulated and 23 were down-regulated compared to the model group (*P* < 0.05). The heat maps illustrating the total metabolites in uterine lavage samples from the model group versus the control group and from the aspirin group versus the model group are shown in (Fig 9.g–h). The heat maps represent the relative expression of metabolites in each group's samples, with red indicating increased biomarker expression and blue indicating decreased biomarker expression. To explore the relationship between potential biomarkers, metabolic pathways, and the mechanisms by which aspirin improves ER in PCOS rats, the metabolites from both the model and control groups, as well as the aspirin and model groups, were intersected. This led to the identification of five common crossover metabolites: 2,6-dihydroxypurine, gluconolactone, Oxaceprol, PC (18:1/18:1), and PC (20:3e/17:1), which were considered potential biomarkers (Fig 9.i and Table 1).

**Table 1:** Potential biomarkers.

Metabolism	Formula	KEGG_ID	HMBD_ID	Model vs Control	Aspirin vs Model				
				P^a^	FC^b^	Trend^c^	P	FC	Trend
PC (20:3e/17:1)	C45H84NO7P	--	--	0.0004^**^	0.45	↓	0.0105^#^	3.15	↑
2,6-dihydroxypurine	C5H4N4O2	--	HMDB0000292	0.0007^**^	0.42	↓	0.0235^#^	2.02	↑
gluconolactone	C6H10O6	--	HMDB0000150	0.0093^**^	0.53	↓	0.0150^#^	1.95	↑
Oxaceprol	C7H11NO4	--	HMDB0000725	0.0213^*^	2.54	↑	0.0233^#^	0.33	↓
PC (18:1/18:1)	C44H84NO8P	--	--	0.0364^*^	0.6	↓	0.0376^#^	1.86	↑

a We applied univariate analysis (t-test) to calculate the statistical significance (P-value).

b The fold change (FC) of relative amounts of the former group compared to the latter group.

c ↑indicates up-regulation; ↓ indicates down-regulation.

*P ＜ 0.05; **P ＜ 0.01 vs. Control group.

# *P* ＜ 0.05 vs. Model group.

#### Metabolic pathway results.

Differential metabolites were imported into the MetaboAnalyst 6.0 platform for pathway enrichment analysis to identify metabolic pathways with *P* < 0.05. Metabolic pathway analysis was performed on the differential metabolites from both the model group compared to the control group and the aspirin group compared to the model group. The results revealed three major metabolic pathways in the model group compared to the control group: the pentose phosphate pathway, arginine biosynthesis, and arginine and proline metabolism (Fig 9.j). In the aspirin group compared to the model group, four major metabolic pathways were identified: purine metabolism, pentose phosphate pathway, glutathione metabolism, and arginine and proline metabolism (Fig 9.k). Notably, the pentose phosphate pathway and arginine and proline metabolism were shared between the control, model, and aspirin groups.

## Discussion

Polycystic ovary syndrome (PCOS) is a common, complex, and heterogeneous endocrine disorder with a multifactorial pathogenesis [15]. It is currently believed to be linked to hyperandrogenemia, insulin resistance, chronic inflammation, and oxidative stress. These pathophysiological changes may contribute to female infertility [2]. PCOS-related infertility accounts for approximately 6%-21% of all infertility cases[17]. The spontaneous abortion rate in PCOS patients is as high as 20%-45%, with recurrent miscarriage rates ranging from 30%-50% [18]. Nearly two-thirds of implantation failures are associated with poor ER [6]. The endometrium is a unique tissue that undergoes cyclic changes under the influence of ovarian steroids, including menstruation, proliferation, secretion, and shedding [19]. A normal endometrial structure and function are essential for promoting embryo implantation and development. Changes in the intrauterine micro-environment can impact ER. ER refers to a series of physiological changes in the endometrium that create an optimal environment for embryo localization, adhesion, invasion, and implantation. A previous study by our team found that aspirin significantly reduced the expression of androgen receptor protein and mRNA, while the expression of progesterone receptor, estrogen receptor, VEGF, and integrin αvβ3 proteins and mRNA were significantly increased in PCOS rats during the window of implantation. This suggests that aspirin may promote angiogenesis through the regulation of VEGF, thereby improving ER [20].

Aspirin is an antiplatelet agent that prevents microthrombus formation by inhibiting platelet aggregation, while simultaneously inhibiting vasoconstriction, leading to improved uterine vasospasm and enhanced ER. Low-dose aspirin (50–150 mg/day) reduces vascular tone and improves tissue perfusion, which results in increased endometrial thickness, glandular development, and activation of multiple estrogen and progesterone receptors. This enhances the uterine response to ovarian hormones, thereby increasing the clinical pregnancy rate [21]. Administration of 50 mg/day of aspirin has been shown to improve endometrial blood perfusion. After two months of treatment with this dose, the endometrial blood flow rate in patients with unexplained recurrent biochemical pregnancy failure significantly increased and even returned to normal levels [22]. Low-dose aspirin also significantly increases the levels of endometrial capacitance proteins, such as leukemia inhibitory factor (LIF) and integrin αvβ3, during the window of implantation [23]. Adequate endometrial blood supply is considered essential for successful implantation. Aspirin irreversibly inhibits cyclooxygenase, reducing the activity of thromboxane A2 (TXA2) and prostaglandins (PGs), which in turn decreases vascular tone by preventing vasoconstriction and improving tissue perfusion, including uterine blood flow velocity [3]. By improving the environment of the endometrium, aspirin facilitates better ER and pregnancy outcomes.

The transformation of the uterus into a receptive state for embryo implantation is primarily regulated by progesterone and estrogen. Additionally, the presence of cytosolic synapses in the endometrium at the capacitated stage is a prerequisite for successful embryo implantation [24]. Numerous studies in rats have demonstrated that the development of cytosolic protrusions is progesterone-dependent and coincides precisely with the implantation window [25]. Cytosolic synapses are critical ultrastructural markers of ER and the implantation window [26]. In the present study, we found that, compared with the model group, the aspirin group exhibited a significant increase in follicle implantation rate, as well as elevated serum levels of estradiol (E_2_) and progesterone (P). Furthermore, endometrial thickness, glandular development, blood vessel formation, increased expression of pinopodes, and an abundance of evenly distributed microvilli were observed, suggesting that aspirin improves sex hormone levels, pinopodes expression, and endometrial morphology in PCOS rats during the window of implantation. These changes contribute to improved ER. This indicates that aspirin can enhance the expression of pinopodes and improve endometrial morphology during the window of implantation in PCOS, which in turn enhances ER and promotes embryo implantation, offering therapeutic potential for treating infertility.

The metabolomics results of the present study indicated that the metabolic profile in the uterine lavage fluid of the PCOS rats during the window of implantation was altered compared with the control group, primarily in the metabolism of metabolites such as 2,6-dihydroxypurine, gluconolactone, Oxaceprol, PC (18:1/18:1), and PC (20:3e/17:1). Aspirin can reverse the changes in the abundance of these five metabolites. Notably, the pentose phosphate pathway and arginine and proline metabolism were the two metabolic pathways shared among the control, model, and aspirin groups.

The pentose phosphate pathway (PPP) is a glucose oxidation pathway that parallels the upper portion of glycolysis, generating ribose 5-phosphate and nicotinamide adenine dinucleotide phosphate (NADPH) [27]. Disruptions in glucose metabolism can impede endometrial glucose utilization, as well as the growth and activity of endometrial cells, potentially leading to endometrial dysplasia, thinning, impaired metaphase, and compromised ER [28]. One critical aspect of establishing ER is the progesterone-mediated differentiation of endometrial stromal cells (ESCs) into decidual cells, which are essential for embryo survival during the early stages of implantation. Dehydroepiandrosterone (DHEA) is an endogenous hormone produced by the adrenal glands, with serum levels elevated approximately twofold in about 50% of patients with PCOS [29]. DHEA is known to be a non-competitive inhibitor of glucose-6-phosphate dehydrogenase (G6PDH), which is the rate-limiting enzyme in the PPP [30]. Frolova proposed that elevated DHEA levels impair implantation by interfering with normal endometrial growth, differentiation, and development into decidual cells, partly due to the inhibition of G6PDH and the resulting blockage of glucose flow through the PPP, which may account for reduced decidualization [29]. The differentiation of ESCs into decidual cells promotes angiogenesis and the maturation of the endometrial vasculature [5]. In the present study, serum metabolites associated with the pentose phosphate pathway, such as gluconolactone, were upregulated after aspirin intervention. Gluconolactone is a polyhydroxy acid (PHA), a lactone or oxidized derivative of glucose. A study has demonstrated that gluconolactone primarily activates extracellular signal-regulated kinase (ERK) signaling through PKCε [31]. ERK, a serine/threonine protein kinase, belongs to the MAPK (mitogen-activated protein kinase) family. Several studies have demonstrated that ERK1/2 phosphorylation (p-ERK1/2) regulates cell proliferation and activity, thereby influencing endometrial receptivity. This kinase plays a critical role in both endometrial epithelial cells (EECs) and stromal cells (ESCs), promoting endometrial cell proliferation, differentiation, and vascular permeability [32]. Upon activation of the ERK1/2-AMPK pathway, the expression of Forkhead Box Protein O1 (FOXO1) is upregulated. Since glucose transporter-1 (GLUT1) is positively regulated by FOXO1, the up-regulation of GLUT1 promotes glucose uptake [33]. This suggests that enhancing the pentose phosphate pathway (PPP) can improve the biosynthesis required for decidualization. Therefore, we hypothesize that the pentose phosphate pathway is one of the mechanisms through which aspirin improves ER in the PCOS rats during the window of implantation.

The arginine and proline metabolic pathway is one of the potential target pathways involved in the development of PCOS [34]. Endocrine dysregulation resulting from chronic inflammation in PCOS may lead to disturbances in vascular homeostasis, contributing to vascular endothelial dysfunction. These disturbances are closely linked to nitric oxide (NO) synthesis, reactive oxygen species, and insulin signaling [35]. Approximately 65%-95% of women with PCOS exhibit insulin resistance and compensatory hyperinsulinemia [36]. L-arginine, a precursor of nitric oxide, is a potent antioxidant that exerts anti-inflammatory effects by scavenging reactive oxygen species [37]. Nitric oxide plays a critical role in vascularization, regulating vascular tone, promoting endometrial metaplasia, mediating angiogenesis, and increasing endometrial vascular permeability and diastolic function. These processes ensure an adequate endometrial blood supply necessary for embryo implantation [38]. The significance of nitric oxide in female reproduction is highlighted by its involvement in follicular maturation, ovulation, decidualization, embryo implantation, and cervical secretion. Increased inducible nitric oxide synthase (iNOS) expression in the uterus during the window of implantation confirms that low nitric oxide levels are associated with reduced uterine tolerance in women with PCOS [39]. Moreover, L-arginine’s ability to lower serum testosterone levels is linked to the binding of nitric oxide to cytochrome P-450, thereby inhibiting steroidogenesis [40]. Arginine, as an intermediate metabolite, connects the tricarboxylic acid cycle to arginine and proline metabolism, suggesting that disturbances in arginine and nitric oxide metabolism may be closely associated with insulin resistance signaling pathways. Studies indicate that insulin signaling is impaired in the pathogenesis of PCOS, potentially due to disruptions in arginine and proline metabolism, leading to nitric oxide production that affects the IRS-1-PI3K/Akt signaling pathway. Evidence suggests that the PI3K/Akt pathway plays a pivotal role in ER by promoting endometrial angiogenesis during the pre-implantation period [41]. Our research results show that the number of endometrial blood vessels increased in the aspirin group. A study has indicated that the metabolism of arginine and proline is reduced in thin endometrial tissues [42]. Our results demonstrate that the endometrium became thinner in the model group, while it thickened in the aspirin group, suggesting that aspirin may increase endometrial thickness by regulating this pathway. Based on these findings, we hypothesize that the arginine and proline metabolic pathways may be the primary metabolic routes through which aspirin improves endometrial receptivity (ER). This improvement is likely achieved by enhancing endometrial blood supply, increasing endometrial thickness, and alleviating insulin resistance, thereby improving endometrial receptivity.

It is reported that PC is predominantly altered in endometrial fluid from implanted and non-implanted IVF cycles, suggesting its importance for ER [43].In our research, in addition to the pentose phosphate pathway and arginine and proline metabolism, two shared metabolites, PC (18:1/18:1) and PC (20:3e/17:1), are noteworthy. Studies have shown that glycerophospholipid metabolism is one of the primary pathways of lipid metabolism. Glycerophospholipids can induce the expression of cyclooxygenase-2 (COX-2), which catalyzes the conversion of arachidonic acid. The metabolites of arachidonic acid are further involved in regulating the gene expression and developmental processes of the endometrium [10]. Glycerophospholipids, such as phosphatidylcholine (PC), are essential components of cell membranes and play pivotal roles in cellular transport, signaling, and the regulation of protein function [44]. Phosphatidylcholine is a primary source of lysophosphatidylcholine (LPC) [45], a lipid that has been linked to inflammatory responses and activation of the phospholipid pathway in insulin resistance. Elevated levels of LPCs suggest a reduced inflammatory response and an improvement in phospholipid metabolism. LPC is an important intermediate in the fatty acid-induced insulin resistance pathway [46]. Women with PCOS often exhibit localized insulin resistance, particularly in the endometrium, where key molecules in the insulin signaling pathway are impaired, leading to disrupted signaling and reduced glucose uptake [21]. LPCs have been shown to promote glucose uptake in adipocytes, with studies on diabetic mouse models suggesting that LPC lowers blood glucose levels by upregulating glucose transporter-4 (GLUT-4) expression on adipocytes, enhancing glucose uptake [47]. GLUT-4 plays a crucial role in facilitating rapid glucose uptake by various cells, maintaining glucose homeostasis. Several studies have reported reduced mRNA and protein levels of GLUT-4 in the endometrium of women with PCOS compared to control individuals, with an even greater reduction observed in women with PCOS and insulin resistance (PCOS-IR). This abnormal expression of GLUT-4 may contribute to endometrial insulin resistance in PCOS patients [48], impairing ER. Relevant studies have shown that abnormal glucose and lipid metabolism is an independent risk factor for insulin resistance (IR), and IR can lead to an increase in testosterone (T) levels in the body, while E_2_ and P levels are suppressed and remain low. Additionally, the study also indicates that E_2_ and P levels are positively correlated with phosphatidylcholine, suggesting that enhancing lipid transport and metabolic capacity plays an important role in restoring hormone levels. The steroid hormones estrogen and progesterone are the main factors controlling uterine receptivity. Estrogen promotes the thickening of the endometrium and increases the number of glands [41], while progesterone significantly enhances the number of pinopodes and promotes their structural integrity[28]. These findings are highly consistent with our research results. Given the importance of the glycerophospholipid metabolic pathway in modulating insulin resistance, we hypothesize that aspirin may improve endometrial insulin sensitivity by regulating this pathway. Through this mechanism, aspirin may enhance ER, potentially contributing to improved fertility outcomes in PCOS patients.

This study focuses on the critical role of glucose metabolism, lipid metabolism, and oxidative stress-related metabolic pathways in the development and progression of PCOS. Based on this finding, we will conduct systematic and in-depth research on the aforementioned three metabolic pathways. Furthermore, we are exploring whether uterine fluid can predict endometrial tolerance in patients with PCOS. If successful, uterine fluid testing may provide a faster and less invasive way for predicting the implantation window and endometrial receptivity. However, several limitations of this study need to be addressed. Firstly, the research lacks experimental validation of key molecules in the relevant metabolic pathways, such as molecular biological evidence from Western blot analysis and immunohistochemical staining. Secondly, due to the small sample size and the fact that only animal experiments were conducted, the generalizability of the research findings requires further verification. Based on these limitations, we plan to carry out follow-up research: on one hand, we will explore the clinical safety and efficacy of aspirin in treating patients with polycystic ovary syndrome (PCOS); on the other hand, we will determine the optimal therapeutic dose of aspirin for improving endometrial receptivity through rigorously designed clinical trials.

## Conclusions

In conclusion, this study provides the first evidence that aspirin modulates glucose metabolism and ameliorates insulin resistance in a PCOS rat model, as revealed by non-targeted metabolic analysis. Specifically, aspirin improves the morphology of the endometrium, promotes endometrial angiogenesis and the development of pinopodes, and ultimately improves embryo implantation rates during the implantation window. These effects are mediated through metabolic pathways in the uterine cavity fluid, including the pentose phosphate pathway, arginine and proline metabolism, and glycerophospholipid metabolism. To further validate these findings, we plan to collect human-derived uterine lavage fluid for multi-sample, multi-dimensional analysis. This will elucidate the mechanisms by which aspirin enhances endometrial receptivity, offering a robust scientific foundation for addressing clinical reproductive challenges.

## Supporting information

S1 Table The number of blastocysts. (XLSX)

S2 Table The levels of E2 and P. (XLSX)

S3 Table Endometrial thickness. (XLSX)

S4 Table Aspirin.vs.Model_Differentially expressed metabolites. (XLSX)

S5 Table Model.vs.Control_Differentially expressed metabolites. (XLSX)

S6 Table Aspirin.vs.Model_pathway_results. (XLSX)

S7 Table Model.vs.Control_pathway_results. (XLSX)

## References

[1] Liu Y, Jiang J-J, Du S-Y, Mu L-S, Fan J-J, Hu J-C, et al. Artemisinins ameliorate polycystic ovarian syndrome by mediating LONP1-CYP11A1 interaction. Science. 2024;384(6701):eadk5382. doi: 10.1126/science.adk5382 38870290

[2] Wang Z, Nie K, Su H, Tang Y, Wang H, Xu X, et al. Berberine improves ovulation and endometrial receptivity in polycystic ovary syndrome. Phytomedicine. 2021;91:153654. doi: 10.1016/j.phymed.2021.153654 34333328

[3] Mourad A, Antaki R, Jamal W, Albaini O. Aspirin for Endometrial Preparation in Patients Undergoing IVF: A Systematic Review and Meta-analysis. J Obstet Gynaecol Can. 2021;43(8):984–992.e2. doi: 10.1016/j.jogc.2021.03.018 33892182

[4] Aref NK, Ahmed WAS, Ahmed MR, Sedik WF. A new look at low-dose aspirin: Co-administration with tamoxifen in ovulation induction in anovulatory PCOS women. J Gynecol Obstet Hum Reprod. 2019;48(8):673–5. doi: 10.1016/j.jogoh.2019.02.004 30807853

[5] Zhang X, Guo F, Wang Q, Bai W, Zhao A. Low-dose aspirin treatment improves endometrial receptivity in the midluteal phase in unexplained recurrent implantation failure. Int J Gynaecol Obstet. 2022;156(2):225–30. doi: 10.1002/ijgo.13699 33829490

[6] Chi Y, He P, Lei L, Lan Y, Hu J, Meng Y, et al. Transdermal estrogen gel and oral aspirin combination therapy improves fertility prognosis via the promotion of endometrial receptivity in moderate to severe intrauterine adhesion. Mol Med Rep. 2018;17(5):6337–44. doi: 10.3892/mmr.2018.8685 29512784 PMC5928622

[7] Giacomini E, Scotti GM, Vanni VS, Lazarevic D, Makieva S, Privitera L, et al. Global transcriptomic changes occur in uterine fluid-derived extracellular vesicles during the endometrial window for embryo implantation. Hum Reprod. 2021;36(8):2249–74. doi: 10.1093/humrep/deab123 34190319 PMC8289330

[8] Monsefi M, Ghasemi A, Alaee S, Aliabadi E. Effects of Anethum graveolens L. (dill) on Oocyte and Fertility of Adult Female Rats. J Reprod Infertil. 2015;16(1):10–7. 25717430 PMC4322175

[9] Abdi A, Ranjbaran M, Amidi F, Akhondzadeh F, Seifi B. The effect of adipose-derived mesenchymal stem cell transplantation on ovarian mitochondrial dysfunction in letrozole-induced polycystic ovary syndrome in rats: the role of PI3K-AKT signaling pathway. J Ovarian Res. 2024;17(1):91. doi: 10.1186/s13048-024-01422-3 38678269 PMC11056058

[10] Liu J, Yang D, Piao C, Wang X, Sun X, Li Y, et al. UPLC-Q-TOF/MS Based Plasma Metabolomics for Identification of Paeonol’s Metabolic Target in Endometriosis. Molecules. 2023;28(2):653. doi: 10.3390/molecules28020653 36677710 PMC9864815

[11] Li L, Xiao Y, Zhou J, Mo H, Li X, Li Y, et al. Effects of Berberine on glucolipid metabolism among dehydroepiandrosterone-induced rats of polycystic ovary syndrome with insulin-resistance. Heliyon. 2024;10(2):e24338. doi: 10.1016/j.heliyon.2024.e24338 38293350 PMC10826177

[12] Ibrahim MAA, Sadek MT, Sharaf Eldin HEM. Role of pomegranate extract in restoring endometrial androgen receptor expression, proliferation, and pinopodes in a rat model of polycystic ovary syndrome. Morphologie. 2022;106(354):145–54. doi: 10.1016/j.morpho.2021.04.004 34023214

[13] Zhang Z, Mu X, Cao Q, Shi Y, Hu X, Zheng H. Honeybee gut Lactobacillus modulates host learning and memory behaviors via regulating tryptophan metabolism. Nat Commun. 2022;13(1):2037. doi: 10.1038/s41467-022-29760-0 35440638 PMC9018956

[14] Wang H, Liu C, Xie X, Niu M, Wang Y, Cheng X, et al. Multi-omics blood atlas reveals unique features of immune and platelet responses to SARS-CoV-2 Omicron breakthrough infection. Immunity. 2023;56(6):1410–1428.e8. doi: 10.1016/j.immuni.2023.05.007 37257450 PMC10186977

[15] Lu C, Zhao X, Li Y, Li Y, Yuan C, Xu F, et al. Serum metabolomics study of Traditional Chinese medicine formula intervention to polycystic ovary syndrome. J Pharm Biomed Anal. 2016;120:127–33. doi: 10.1016/j.jpba.2015.12.020 26730509

[16] Yao D, Shen C, Zhang X, Tang J, Yu J, Tu M, et al. Untargeted metabolomics study of mature human milk from women with and without gestational diabetes mellitus. Food Chem. 2024;460(Pt 3):140663. doi: 10.1016/j.foodchem.2024.140663 39142199

[17] Naqvi SMAS, Bhattarai JB, Li H, Wang XW. Polycystic Ovarian Syndrome and Female Infertility. YM. 2020;04(01):11–27. doi: 10.4236/ym.2020.41002

[18] Bui LM, Aghajanova L, Lathi RB, Sokalska A. Polycystic ovary syndrome and miscarriage: a narrative review. F&S Reviews. 2024;5(4):100078. doi: 10.1016/j.xfnr.2024.100078

[19] Puente E, Alonso L, Laganà AS, Ghezzi F, Casarin J, Carugno J. Chronic Endometritis: Old Problem, Novel Insights and Future Challenges. Int J Fertil Steril. 2020;13(4):250–6. doi: 10.22074/ijfs.2020.5779 31710184 PMC6875860

[20] Liu Q, He YQ, Zhang Y, Wang X, Yang S. Investigation of mechanism of endometrial tolerance during the “window of implantation” in PCOS rats regulated by cyclic therapy based on miR-140-5p/VEGF pathway. Chinese Journal of Experimental Traditional. 1–10. doi: 10.13422/j.cnki.syfjx.20242041

[21] Zhao Y, Du B, Jiang X, Ma M, Shi L, Zhang Q, et al. Effects of combining low‑dose aspirin with a Chinese patent medicine on follicular blood flow and pregnancy outcome. Mol Med Rep. 2014;10(5):2372–6. doi: 10.3892/mmr.2014.2570 25230733

[22] Zhang X, Guo F, Wang Q, Bai W, Zhao A. Low-dose aspirin improves blood perfusion of endometrium of unexplained recurrent biochemical pregnancy loss. Int J Gynaecol Obstet. 2022;157(2):418–23. doi: 10.1002/ijgo.13838 34314517

[23] Zhao M, Chang C, Liu Z, Chen LM, Chen Q. Treatment with low-dose aspirin increased the level LIF and integrin β3 expression in mice during the implantation window. Placenta. 2010;31(12):1101–5. doi: 10.1016/j.placenta.2010.10.002 21035850

[24] Aunapuu M, Kibur P, Järveots T, Arend A. Changes in Morphology and Presence of Pinopodes in Endometrial Cells during the Luteal Phase in Women with Infertility Problems: A Pilot Study. Medicina (Kaunas). 2018;54(5):69. doi: 10.3390/medicina54050069 30344300 PMC6262557

[25] Nikas G, Aghajanova L. Endometrial pinopodes: some more understanding on human implantation?. Reprod Biomed Online. 2002;4:18–23. doi: 10.1016/s1472-6483(12)60111-4 12470560

[26] Rarani FZ, Borhani F, Rashidi B. Endometrial pinopode biomarkers: Molecules and microRNAs. J Cell Physiol. 2018;233(12):9145–58. doi: 10.1002/jcp.26852 29968908

[27] TeSlaa T, Ralser M, Fan J, Rabinowitz JD. The pentose phosphate pathway in health and disease. Nat Metab. 2023;5(8):1275–89. doi: 10.1038/s42255-023-00863-2 37612403 PMC11251397

[28] Bai X, Zheng L, Li D, Xu Y. Research progress of endometrial receptivity in patients with polycystic ovary syndrome: a systematic review. Reprod Biol Endocrinol. 2021;19(1):122. doi: 10.1186/s12958-021-00802-4 34362377 PMC8344130

[29] Frolova AI, O’Neill K, Moley KH. Dehydroepiandrosterone inhibits glucose flux through the pentose phosphate pathway in human and mouse endometrial stromal cells, preventing decidualization and implantation. Mol Endocrinol. 2011;25(8):1444–55. doi: 10.1210/me.2011-0026 21680659 PMC3146244

[30] Jimenez PT, Frolova AI, Chi MM, Grindler NM, Willcockson AR, Reynolds KA, et al. DHEA-mediated inhibition of the pentose phosphate pathway alters oocyte lipid metabolism in mice. Endocrinology. 2013;154(12):4835–44. doi: 10.1210/en.2012-2140 24036000 PMC3836065

[31] Qin X, Liu B, Gao F, Hu Y, Chen Z, Xu J, et al. Gluconolactone Alleviates Myocardial Ischemia/Reperfusion Injury and Arrhythmias via Activating PKCε/Extracellular Signal-Regulated Kinase Signaling. Front Physiol. 2022;13:856699. doi: 10.3389/fphys.2022.856699 35360251 PMC8964113

[32] Wang B, Gao M, Yao Y, Shen H, Li H, Sun J, et al. Enhancing endometrial receptivity: the roles of human chorionic gonadotropin in autophagy and apoptosis regulation in endometrial stromal cells. Reprod Biol Endocrinol. 2024;22(1):37. doi: 10.1186/s12958-024-01205-x 38576003 PMC10993617

[33] Huang J, Xue M, Zhang J, Yu H, Gu Y, Du M, et al. Protective role of GPR120 in the maintenance of pregnancy by promoting decidualization via regulation of glucose metabolism. EBioMedicine. 2019;39:540–51. doi: 10.1016/j.ebiom.2018.12.019 30578080 PMC6355327

[34] Murata H, Tanaka S, Okada H. The Regulators of Human Endometrial Stromal Cell Decidualization. Biomolecules. 2022;12(9):1275. doi: 10.3390/biom12091275 36139114 PMC9496326

[35] Huang W, Li S, Luo N, Lu K, Ban S, Lin H. Dynamic Analysis of the Biochemical Changes in Rats with Polycystic Ovary Syndrome (PCOS) Using Urinary 1H NMR-Based Metabonomics. Horm Metab Res. 2020;52(1):49–57. doi: 10.1055/a-1073-2346 31945791

[36] Ezeh U, Pisarska MD, Azziz R. Association of severity of menstrual dysfunction with hyperinsulinemia and dysglycemia in polycystic ovary syndrome. Hum Reprod. 2022;37(3):553–64. doi: 10.1093/humrep/deac001 35048126 PMC8888996

[37] Tian J, Xu Y, Xiong Y, Zuo L, Zhou M, Cao C, et al. Metabolomics combined with network pharmacology to explore the mechanisms of modified Guishen pill to ameliorate polycystic ovary syndrome. Comput Biol Med. 2022;148:105790. doi: 10.1016/j.compbiomed.2022.105790 35839542

[38] Shen M, Liu Y, Ma X, Zhu Q. Erbu Zhuyu decoction improves endometrial angiogenesis via uterine natural killer cells and the PI3K/Akt/eNOS pathway a mouse model of embryo implantation dysfunction. Am J Reprod Immunol. 2023;89(1):e13634. doi: 10.1111/aji.13634 36327113 PMC10078112

[39] Krishna MB, Joseph A, Thomas PL, Dsilva B, Pillai SM, Laloraya M. Impaired Arginine Metabolism Coupled to a Defective Redox Conduit Contributes to Low Plasma Nitric Oxide in Polycystic Ovary Syndrome. Cell Physiol Biochem. 2017;43(5):1880–92. doi: 10.1159/000484107 29055959

[40] Li J, Zhu H, Zhu Y, Wu P, Wu H, Chen H. Effect of Bushen Huoxue recipe on serum metabolomics in polycystic ovary syndrome rats. Gynecol Endocrinol. 2023;39(1):2260500. doi: 10.1080/09513590.2023.2260500 37849277

[41] Yusuf ANM, Amri MF, Ugusman A, Hamid AA, Wahab NA, Mokhtar MH. Hyperandrogenism and Its Possible Effects on Endometrial Receptivity: A Review. Int J Mol Sci. 2023;24(15):12026. doi: 10.3390/ijms241512026 37569402 PMC10419014

[42] Xu L, Fan Y, Wang J, Shi R. Dysfunctional intercellular communication and metabolic signaling pathways in thin endometrium. Front Physiol. 2022;13:1050690. doi: 10.3389/fphys.2022.1050690 36505055 PMC9729336

[43] Tian J, Zhang Z, Mei J, Kong N, Yan Y, Shen X, et al. Dysregulation of endometrial stromal serotonin homeostasis leading to abnormal phosphatidylcholine metabolism impairs decidualization in patients with recurrent implantation failure. Hum Reprod Open. 2024;2024(3):hoae042. doi: 10.1093/hropen/hoae042 39091587 PMC11293872

[44] Matsuyama S, Whiteside S, Li S-Y. Implantation and Decidualization in PCOS: Unraveling the Complexities of Pregnancy. Int J Mol Sci. 2024;25(2):1203. doi: 10.3390/ijms25021203 38256276 PMC10816633

[45] Guan S-Y, Liu Y-Y, Guo Y, Shen X-X, Liu Y, Jin H-X. Potential biomarkers for clinical outcomes of IVF cycles in women with/without PCOS: Searching with metabolomics. Front Endocrinol (Lausanne). 2022;13:982200. doi: 10.3389/fendo.2022.982200 36120473 PMC9478024

[46] Han MS, Lim Y-M, Quan W, Kim JR, Chung KW, Kang M, et al. Lysophosphatidylcholine as an effector of fatty acid-induced insulin resistance. J Lipid Res. 2011;52(6):1234–46. doi: 10.1194/jlr.M014787 21447485 PMC3090244

[47] Liu P, Zhu W, Chen C, Yan B, Zhu L, Chen X, et al. The mechanisms of lysophosphatidylcholine in the development of diseases. Life Sci. 2020;247:117443. doi: 10.1016/j.lfs.2020.117443 32084434

[48] Li J, Chen S, Qin R, Liu X, Fan L, Wei M, et al. Talin1 regulates glucose metabolism and endometrial receptivity via GLUT-4 in patients with polycystic ovary syndrome and insulin resistance. Gynecol Endocrinol. 2023;39(1):2231085. doi: 10.1080/09513590.2023.2231085 37395213

